# The Treatment of Physical Pain

**Published:** 1892-12

**Authors:** 


### The Treatment of Physical Pain.

Professor Hayem (*Journal of Nervous and Mental Diseases*) in an article on this subject gives the results of some very interesting studies in this direction. He contends that to intelligently treat pain the varieties must be thoroughly understood, and, to facilitate matters, classifies pain as follows: Class 1, treated according to the intensity; Class 2, according to location; Class 3, the course of the pain, as to periodicity, duration, etc.; Class 4, the age of the case.

The intensity of the pain is of great importance when it comes to treatment, for pain can be of such severity and frequency as to cause death; for instance, pain of kidney and liver colic, and of angina pectoris. From the frequency with which certain cases have paroxysms of pain, it is evident that such remedies as chloroform and morphine can not be constantly employed, and it is in this class of cases that the ingenuity of the physician is most severely taxed. One of the most painful affections is facial neuralgia; the remedies mostly employed are aconitine, morphine, and atropine hypodermically; antipyrine is sometimes used in the same manner. For internal medication, quinine, antifebrin and exalgine seem to offer the best results, the quinine to be given during the interval between the paroxysms. Aconitine is the drug mostly relied upon to control the pain; when the suffering is not severe, the antifebrin and exalgine are employed with benefit. Where the neuralgia is of the trunk or extremities, some remedy must be used which acts locally, such as revulsives. One of the best remedies which the author has found is the refrigeration of the part by chlormethyl. If the condition is of the congestive form, as in recent rheumatic neuralgia, scarification is good practice. The topical application of a sedative is sometimes followed by good results. Some of the alkaloids of opium, made up in an ointment, to be rubbed over the part, often relieve pain. Veratrine, camphor, and menthol can be used in this way. In hemicrania, dependent upon bad digestion, antipyrin and phenacetin, internally administered, is often followed by relief from pain.

The opium preparations alone, or in combination with cocaine, are particularly indicated in the smarting pain occurring in neuroses of the digestive tract. The so-called rheumatic neuralgia is best controlled by the internal administration of quinine and salicylate of sodium.

